# Vaccination coverage in Italian children and antimicrobial resistance: an ecological analysis

**DOI:** 10.1186/s13756-022-01173-0

**Published:** 2022-11-09

**Authors:** Andrea Maugeri, Martina Barchitta, Antonella Agodi

**Affiliations:** 1 Department of Medical and Surgical Sciences and Advanced Technologies “GF Ingrassia”, University of Catania, Via S. Sofia 87, 95123 Catania, Italy; 2 Azienda Ospedaliero Universitaria Policlinico “G. Rodolico-San Marco”, Via S. Sofia 78, 95123, Catania, Italy

**Keywords:** Antibiotic, Vaccine, Antimicrobial use, Infections

## Abstract

**Background:**

Although a general consensus that vaccines could be a complementary strategy against antimicrobial resistance (AMR), there is still the need for studies investigating the relationship between childhood vaccination coverage and AMR proportions in the overall population.

**Methods:**

We performed an ecological analysis of available Italian data (vaccination coverages, AMR proportions, number of isolates tested, and antibiotic use) to evaluate the relationships between vaccination coverages in children and AMR proportions in the last 2 decades.

**Results:**

After adjusting for covariates, we showed that AMR proportions decreased with increasing vaccination coverages, especially for some combinations of vaccines, pathogens, and antimicrobials. Vaccination coverages for pertussis, diphtheria, and tetanus were inversely related to proportions of *E. coli* resistant to fluoroquinolones and third generation cephalosporins, *K. pneumoniae* resistant to carbapenems and third generation cephalosporins, and *P. aeruginosa* resistant to piperacillin and tazobactam. Polio vaccination coverage was inversely related to proportions of *E. coli* and *K. pneumoniae* resistant to third generation cephalosporins.

**Conclusions:**

These results, however, should be interpreted cautiously due to the ecological nature of our analysis. For this reason, further studies designed ad hoc should be encouraged to measure the impact of increasing childhood vaccination coverage on AMR.

**Supplementary Information:**

The online version contains supplementary material available at 10.1186/s13756-022-01173-0.

## Background

Antimicrobial resistance (AMR) continues to constitute a significant issue for public health. According to the first report published jointly by the European Centre for Disease Prevention and Control (ECDC) and the WHO Regional Office for Europe, in fact, there were approximately 670,000 antimicrobial-resistant infections and 33,000 deaths in the EU/EEA in 2020 [1]. This results in an economic impact that was estimated around €1.5 billion [2]. Although frequency and distribution of AMR vary across Europe, Italy has one of the highest incidences [1]. Italy, in fact, shows both high incidence of healthcare-associated infections [3–6] and high proportions of *Escherichia coli*, *Klebsiella pneumoniae*, and *Staphylococcus aureus* strains resistant to antimicrobials [1]. Considering this scenario, current preventive measures and actions are crucial to counteract the spread of antimicrobial-resistant pathogens [7]. Nevertheless, in line with the latest forecasts, the number of people dying as a direct consequence of antimicrobial-resistant infections will exceed those due to cancer by 2050 [8]. The reality is that no single response is sufficient for curbing the growing tide of AMR. The development of new antibiotics and other forms of treatment (e.g., monoclonal antibodies) could provide options to ensure that some pathogens remain responsive to some form of treatment. The improvement of regulation of access to antimicrobials in human and animal populations, as well as better programs of antibiotic stewardship, could help preserve the effectiveness of current existing therapies [9]. For all these reasons, alternative and multisectoral strategies should be first explored and then introduced to help the fight against AMR.

There is no doubt that vaccination can play a complementary role in combating AMR, but its application has been largely underestimated to date. Yet, vaccines could counteract AMR by reducing the incidence of infections caused by susceptible or resistant pathogens, viruses, fungi and parasites [10]. Indeed, the latter are often inappropriately treated with antibiotics. This hypothesis is documented and supported by previous real-world examples showing how both bacterial and viral vaccines might have an impact on AMR [10, 11]. Some vaccines are under development (e.g., those for Group B Streptococcus and Staphylococci) or already in use to directly tackle AMR (e.g., those against *Hemophilus influenzae* Type B and Pneumococcus) [12]. Others such as viral vaccines, instead, could counteract AMR through an indirect way, by reducing the inappropriate use of antimicrobials for viral infections [10, 11, 13]. In a previous study, for instance, we evaluated the relationship between influenza vaccination and AMR in Italy, showing that higher vaccination coverage corresponded to lower AMR proportions, especially for *Escherichia coli* and *Klebsiella pneumoniae* [14].

Taken together, these results generated broad consensus on the potential role of vaccination in fighting AMR. However, current evidence is generally vague due to the difficulties of measuring the magnitude and direct effect of vaccines on AMR. Different study designs can be applied to evaluate the efficacy and effectiveness of vaccination on AMR. Even though intervention studies produce the highest level of evidence, rarely they are specifically designed to focus on AMR. At most, changes in AMR proportion are assessed as part of the overall vaccine evaluation. Ecological studies, instead, are important to monitor AMR proportions over the years, and to evaluate their relationship with vaccination coverages. The results thus obtained could be useful to generate new hypotheses and then confirm them through a multi-level approach. In particular, there is still a need for assessing whether childhood vaccination might be—at least in part—related to AMR in the general population. With this rationale, the present ecological study aimed to evaluate the relationship between vaccination coverages in children and AMR proportions over the last 2 decades in Italy.

## Methods

### Data on vaccination coverages

We used data on vaccination coverages in children (≤ 24 months), from 2000 to 2020 in Italy. This information, expressed as the proportion of vaccinated subjects per 100 inhabitants, was reported by the Italian National Institute of Health (Istituto Superiore di Sanità, ISS) and made publicly available on the website www.epicentro.iss.it/vaccini/dati_Ita. These data are routinely collected through immunization registries implemented at regional level and then transmitted at national level [15]. The current analysis was conducted on the following vaccinations:

- Pertussis vaccine
- Diphtheria vaccine
- Tetanus vaccine
- Polio vaccine
- *Haemophilus influenzae* Type B (Hib) vaccine
- Measles, Mumps, and Rubella (MMR) vaccine
- Varicella vaccine

It is worth mentioning that data on vaccination coverage for pertussis, diphtheria, and tetanus vaccines were reported in the aggregate (diphtheria-tetanus-pertussis vaccine; DTaP) until 2013, and then by specific antigen. Since there were slight differences in vaccination coverages for specific antigens, we conducted separated analyses for pertussis, diphtheria, and tetanus vaccines. Even the data on vaccines against measles, mumps, and rubella were initially reported in the aggregate and then by specific antigen. However, there were no differences in specific vaccination coverages and thus we analyzed data for the MMR vaccine (for additional details, these data and their visualizations are publicly available on the website www.epicentro.iss.it/vaccini/dati_Ita).

### Data on AMR proportions

We also used data on AMR proportions in Italy, provided by the Italian AMR surveillance project of the ISS (AR-ISS) for selected isolates and specific periods throughout the Italian country. The AR-ISS, based on a network of local clinical laboratories testing the antimicrobial susceptibility of invasive isolates of eight key bacterial species, reports AMR proportions to the to the European Antimicrobial Resistance Surveillance Network (EARS-Net) of the ECDC [16]. These data are made publicly available on the Surveillance Atlas of Infectious Diseases of the ECDC [17]. It is worth mentioning that the number of sentinel laboratories increased over the years in Italy, from 62 in 2001 to 153 in 2021. The latter figure corresponded to a national coverage of 47.3% [18]. In the current analysis, we used data on the number of tested isolates and AMR proportions for the following combinations of pathogens and antimicrobials, included in the Italian and European surveillance networks:

- *E. coli* resistant to fluoroquinolones, third generation cephalosporins, aminoglycosides, or aminopenicillins;
- *K. pneumoniae* resistant to carbapenems, third generation cephalosporins, aminoglycosides, or fluoroquinolones;
- *Acinetobacter baumannii* resistant to aminoglycosides, carbapenems, or fluoroquinolones;
- *Pseudomonas aeruginosa* resistant to fluoroquinolones, piperacillin and tazobactam, carbapenems, or ceftazidime;
- *S. aureus* resistant to methicillin (MRSA);
- *Streptococcus pneumoniae* resistant to penicillins or macrolides;
- *Enterococcus faecium* resistant to aminopenicillins, vancomycin, or gentamicin;
- *Enterococcus faecalis* resistant to aminopenicillins, vancomycin, or gentamicin.

### Data on antimicrobial use

Data on antimicrobial use in the community were obtained from the European Surveillance of Antimicrobial Consumption Network (ESAC-Net) database [19]. The ESAC-Net collects data on the consumption of antimicrobials for systemic use through the European Surveillance System (TESSy), according to the Anatomical Therapeutic Chemical (ATC) classification system for the allocation of antimicrobials in groups. Antibiotic consumption is reported as the number of Defined Daily Doses (DDDs) per 1000 inhabitants per day, applying the latest ATC/DDD index in all years. For Italy, this information is based on sales and reimbursement data [19]. In the current analysis, we used data on the consumption of the following antimicrobials:

- Fluoroquinolones (J01MA);
- Carbapenems (J01DH);
- Third generation cephalosporins (J01DD);
- Combinations of penicillins (J01CR);

### Statistical analysis

We first used summary statistics (i.e., mean, standard deviation SD, and range) to describe vaccination coverages, AMR proportions, and antibiotic use in Italy. Next, we evaluated correlations between annual vaccination coverages and AMR proportions, using the Spearman's rank correlation coefficient (rho). Correlation coefficients were displayed as a heat-map for all combinations of vaccine, pathogen, and resistance to antimicrobials. For combinations showing statistically significant correlations after Bonferroni correction for 168 multiple comparisons (adjusted-α = 0.0003), we applied linear regression analyses to evaluate the relationship between vaccination coverage (independent variable) and AMR proportion (dependent variable), adjusting for the number of isolates tested and antimicrobial use. The latter data were obtained from the Italian National Institute of Statistics (Istituto Nazionale di Statistica, ISTAT) [20]. Outputs of linear regressions were expressed as β coefficient and its standard error (SE). All the analyses were performed on the SPSS software (version 23.0, SPSS, Chicago, IL, USA), with a significance level α of 0.05 unless otherwise stated.

## Results

Overall, the average vaccination coverages were 95.5% (SD = 1.1; range 93.3–96.8) for pertussis vaccine, 95.6% (SD = 1.1; range 93.4–96.8) for diphtheria vaccine, 95.7% (SD = 1.0; range 93.6–96.8%) for tetanus vaccine, 95.6% (SD = 1.1; range 93.3–96.8%) for polio vaccine, 90.9% (SD = 10.1; range 54.7–96.0%) for Hib vaccine, 87.5% (SD = 5.1; range 74.1–94.4%) for MMR vaccine, and 74.5% (SD = 21.5; range 30.7–90.6%) for varicella vaccine, with some variations from 2000 to 2020. While pertussis, diphtheria, tetanus, and polio vaccination coverages slightly decreased over the years, Hib and MMR vaccination coverages increased. No statistically significant trend was evident for varicella vaccination coverage. More details on trends of vaccination coverages are reported in the Additional file 1, as well as on the website www.epicentro.iss.it/vaccini/dati_Ita. With respect to AMR, the overall number of isolates tested ranged from 6014 for *S. pneumoniae* to 58,468 for *S. aureus*. In general, the number of isolates tested annually increased over the years. AMR proportions exhibited peculiar trends over the years, also in relation to different pathogen-antimicrobial combinations considered. More details on trends of AMR proportions are reported in the Additional file 2, as well as in the Surveillance Atlas of Infectious Diseases of the ECDC.

**Fig. 1:**
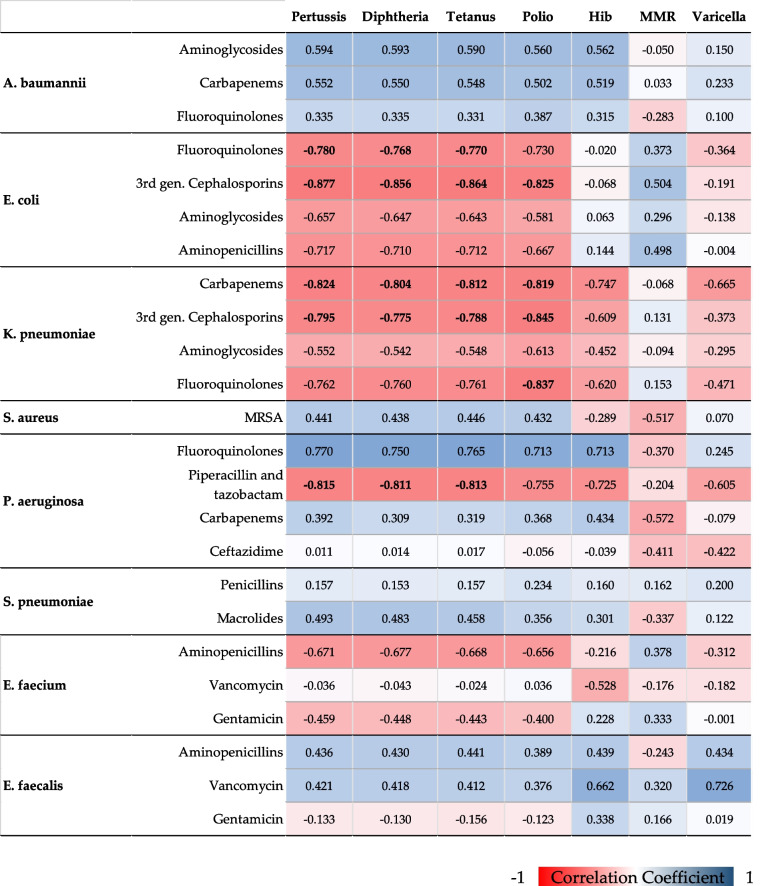
Spearman's Correlations between vaccination coverages and antimicrobial resistances. Correlations coefficients with *p* < 0.0003 (Bonferroni-adjusted α) are indicated in bold font

Figure 1 summarizes Spearman's correlation coefficients between vaccination coverage and AMR proportion for each combination of vaccine, pathogen, and antimicrobial. Pertussis vaccination coverage inversely correlated with proportions of: *E. coli* resistant to fluoroquinolones (rho =  − 0.780) and third generation cephalosporins (rho =  − 0.877; *K. pneumoniae* resistant to carbapenems (rho =  − 0.824) and third generation cephalosporins (rho =  − 0.795); *P. aeruginosa* resistant to piperacillin and tazobactam (rho =  − 0.815). As expected, we also found similar correlation coefficients when analyzing the relationship between AMR proportions and coverages of diphtheria and tetanus vaccines. Polio vaccination coverage exhibited a similar pattern of correlation with the different combinations of pathogen and antimicrobial. In fact, it inversely correlated with proportions of: *E. coli* resistant to third generation cephalosporins (rho =  − 0.825); *K. pneumoniae* resistant to carbapenems (rho =  − 0.819), third generation cephalosporins (rho =  − 0.845), and fluoroquinolones (rho =  − 0.837). Vaccination coverages for Hib, MMR and Varicella, instead, showed less marked correlations with AMR proportions, which were not significant after Bonferroni correction.

Next, we tested the linear relationship between vaccination coverage and AMR proportion for each combination of vaccine, pathogen, and resistance to antimicrobials with statistically significant correlation.

**Fig. 2:**
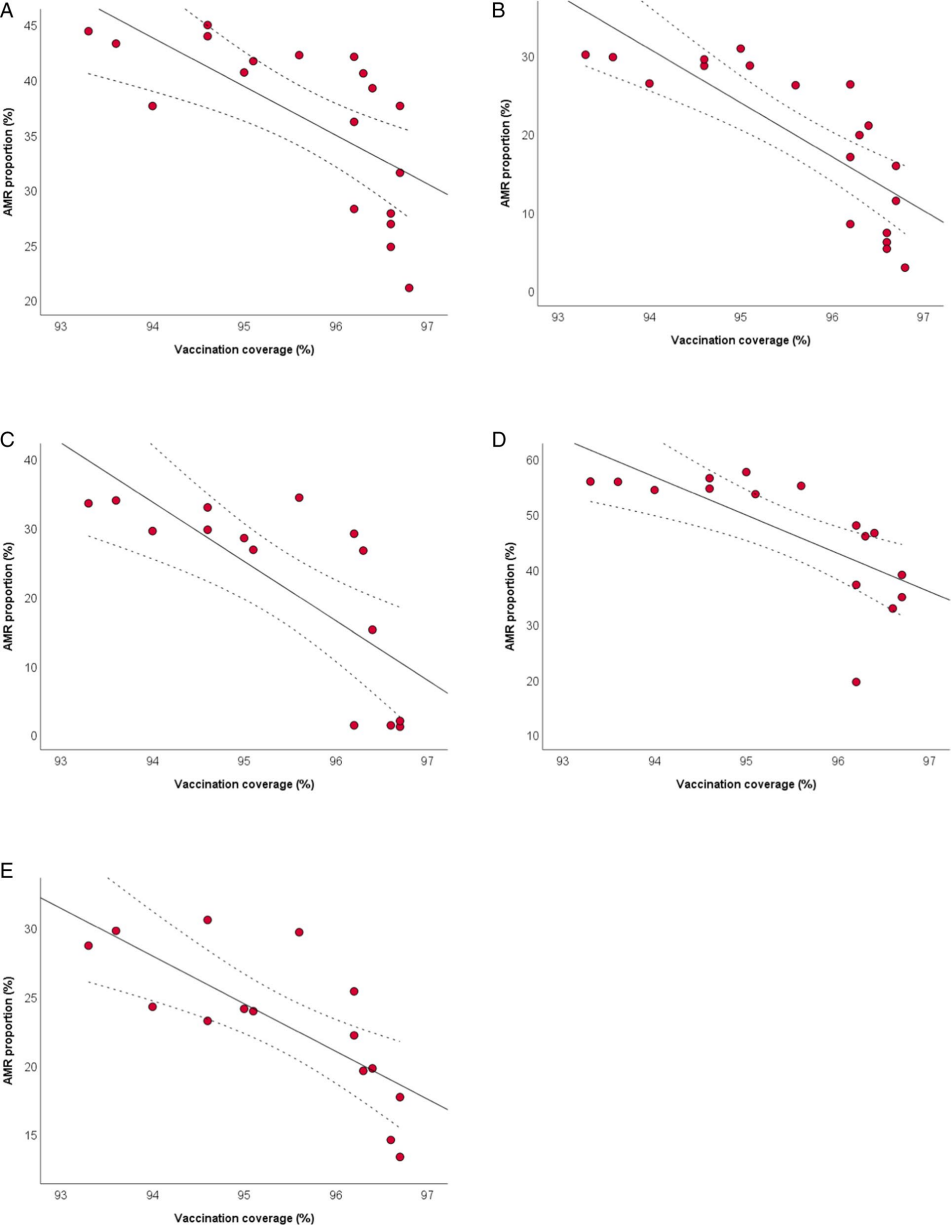
The relationship between pertussis vaccination coverage and AMR. The relationships with proportions of *Escherichia coli* resistant to fluoroquinolones (**A**) and third generation cephalosporins (**B**); *K. pneumoniae* resistant to carbapenems (**C**) and third generation cephalosporins (**D**); *P. aeruginosa* resistant to piperacillin and tazobactam (**E**). The plots show linear regression lines and their 95%CI (dotted lines)

Figure 2 shows the scatter plots of the inverse relationships of pertussis vaccination coverage with AMR proportions of *E. coli*, *K. pneumoniae*, and *P*. aeruginosa. After adjusting for the number of isolates tested and for antimicrobial use, the analysis confirmed the inverse relationships of pertussis vaccination coverage with the proportions of *E. coli* resistant to fluoroquinolones (β =  − 2.823; SE = 1.176; *p* = 0.030) and third generation cephalosporins (β =  − 4.620; SE = 1.416; *p* = 0.005), as well as with the proportions of *K. pneumoniae* resistant to carbapenems (β =  − 4.247; SE = 1.767; *p* = 0.038) and third generation cephalosporins (β =  − 5.456; SE = 2.170; *p* = 0.027). The adjusted analysis also confirmed the inverse relationship of pertussis vaccination coverage with the proportion of *P. aeruginosa* resistant to piperacillin and tazobactam (β =  − 4.131; SE = 0.974; *p* = 0.001). All the results obtained by applying linear regression models are reported in the Additional file 3.

**Fig. 3:**
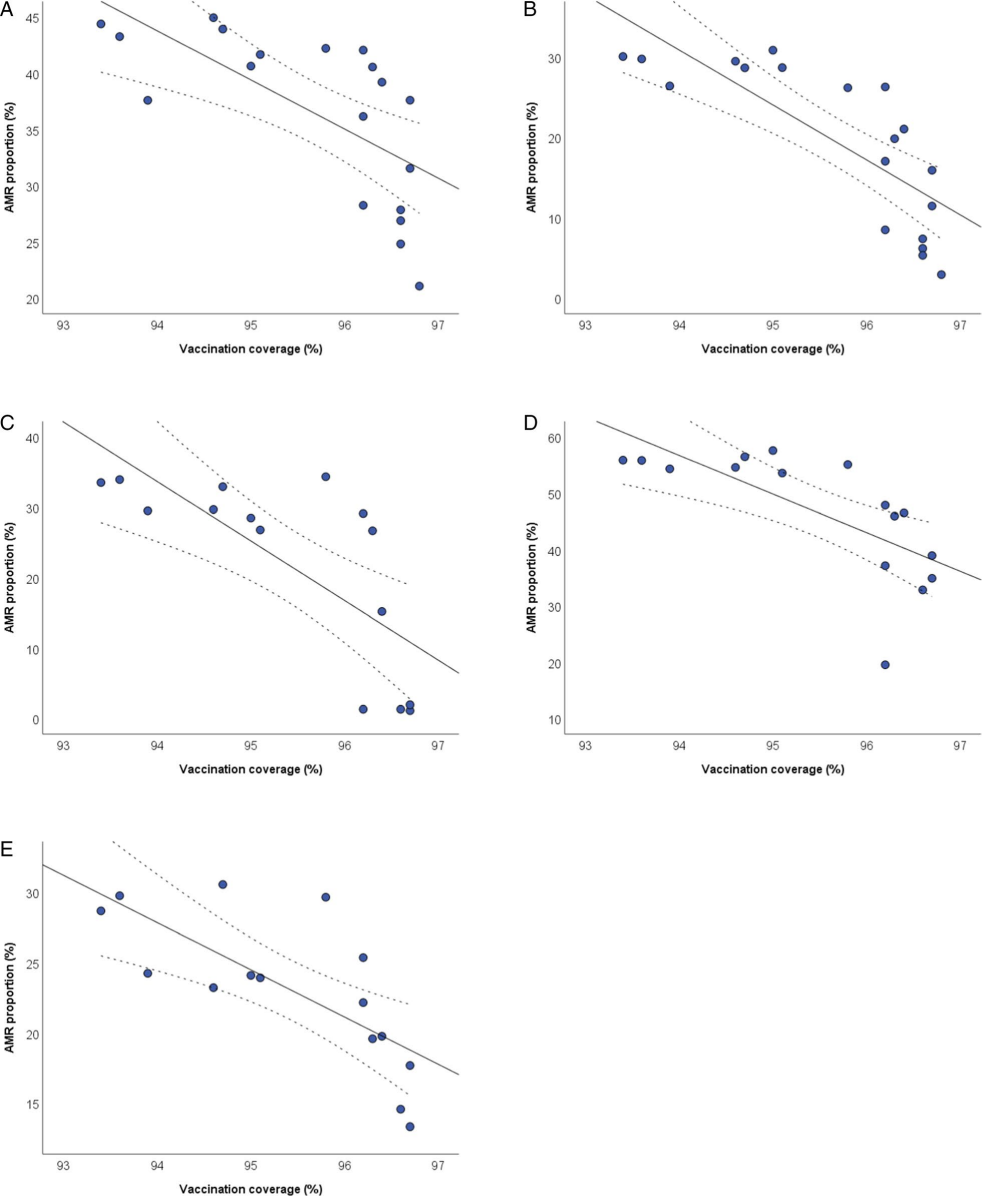
The relationship between diphtheria vaccination coverage and AMR. The relationships with proportions of *Escherichia coli* resistant to fluoroquinolones (**A**) and third generation cephalosporins (**B**); *K. pneumoniae* resistant to carbapenems (**C**) and third generation cephalosporins (**D**); *P. aeruginosa* resistant to piperacillin and tazobactam (**E**). The plots show linear regression lines and their 95%CI (dotted lines)

Similar results were obtained for the diphtheria and tetanus vaccines, since data on vaccination coverage showed only minor differences with pertussis vaccine. Figure 3 shows the scatter plots of the inverse relationships of diphtheria vaccination coverage with AMR proportions of *E. coli*, *K. pneumoniae*, and *P*. aeruginosa. After adjusting for the number of isolates tested and antimicrobial use, diphtheria vaccination coverage was inversely related to the proportions of: *E. coli* resistant to fluoroquinolones (β =  − 2.856; SE = 1.190; *p* = 0.030) and third generation cephalosporins (β =  − 4.648; SE = 1.450; *p* = 0.006); *K. pneumoniae* resistant to carbapenems (β =  − 4.734; SE = 2.080; *p* = 0.039) and third generation cephalosporins (β =  − 5.404; SE = 2.258; *p* = 0.034); *P. aeruginosa* resistant to piperacillin and tazobactam (β =  − 3.885; SE = 1.085; *p* = 0.004). All the results obtained by applying linear regression models are reported in the Additional file 4.

**Fig. 4:**
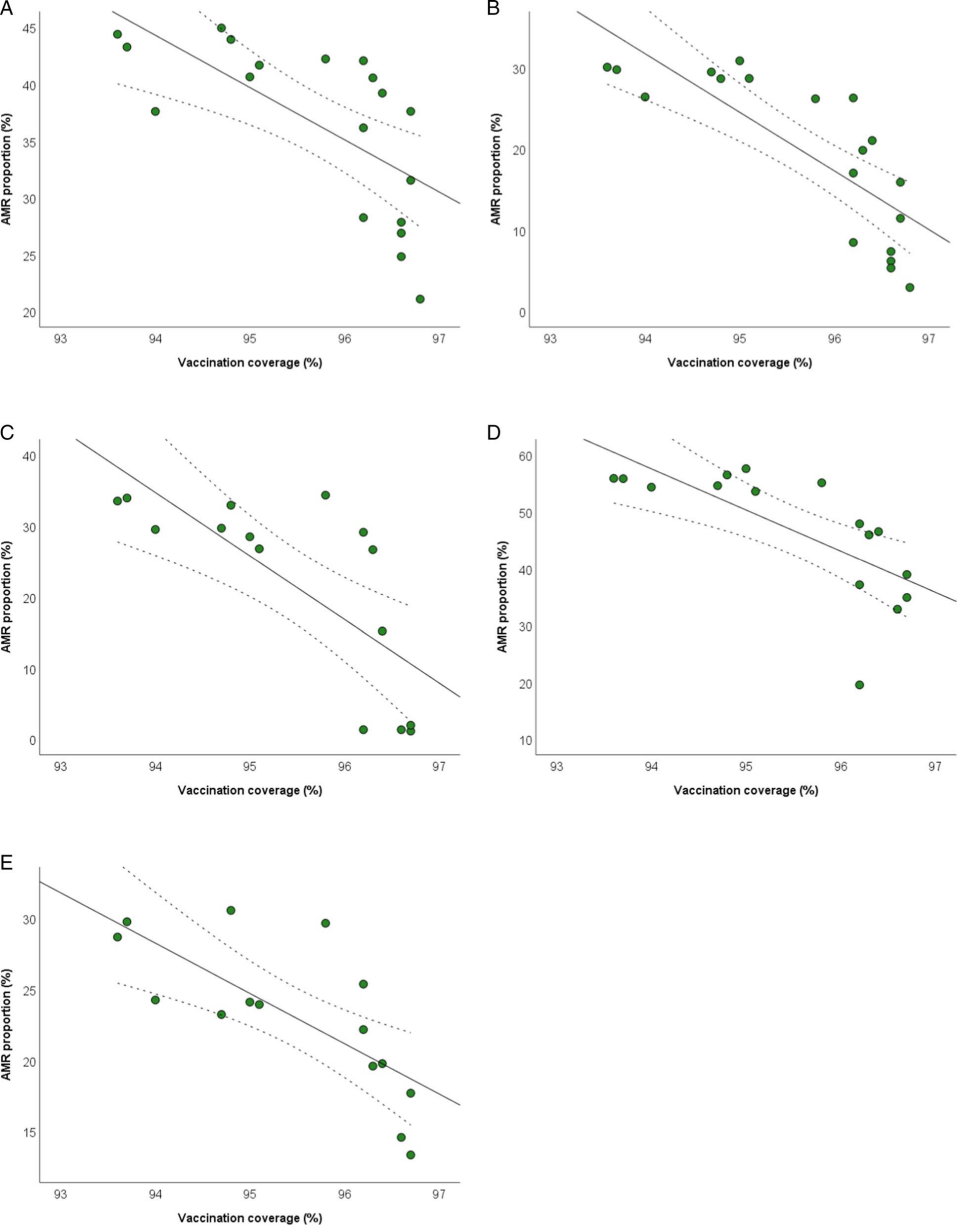
The relationship between tetanus vaccination coverage and AMR. The relationships with proportions of *Escherichia coli* resistant to fluoroquinolones (**A**) and third generation cephalosporins (**B**); *K. pneumoniae* resistant to carbapenems (**C**) and third generation cephalosporins (**D**); *P. aeruginosa* resistant to piperacillin and tazobactam (**E**). The plots show linear regression lines and their 95%CI (dotted lines)

Figure 4 shows the scatter plots of the inverse relationships of tetanus vaccination coverage with AMR proportions of *E. coli*, *K. pneumoniae*, and *P*. aeruginosa. In particular, tetanus vaccination coverage was inversely related to the proportions of: *E. coli* resistant to fluoroquinolones (β =  − 3.059; SE = 1.260; *p* = 0.028) and third generation cephalosporins (β =  − 4.944; SE = 1.538; *p* = 0.006); *K. pneumoniae* resistant to carbapenems (β =  − 4.380; SE = 2.095; *p* = 0.043) and third generation cephalosporins (β =  − 5.744; SE = 2.403; *p* = 0.033); *P. aeruginosa* resistant to piperacillin and tazobactam (β =  − 4.184; SE = 1.138; *p* = 0.003). All the results obtained by applying linear regression models are reported in the Additional file 5.

**Fig. 5:**
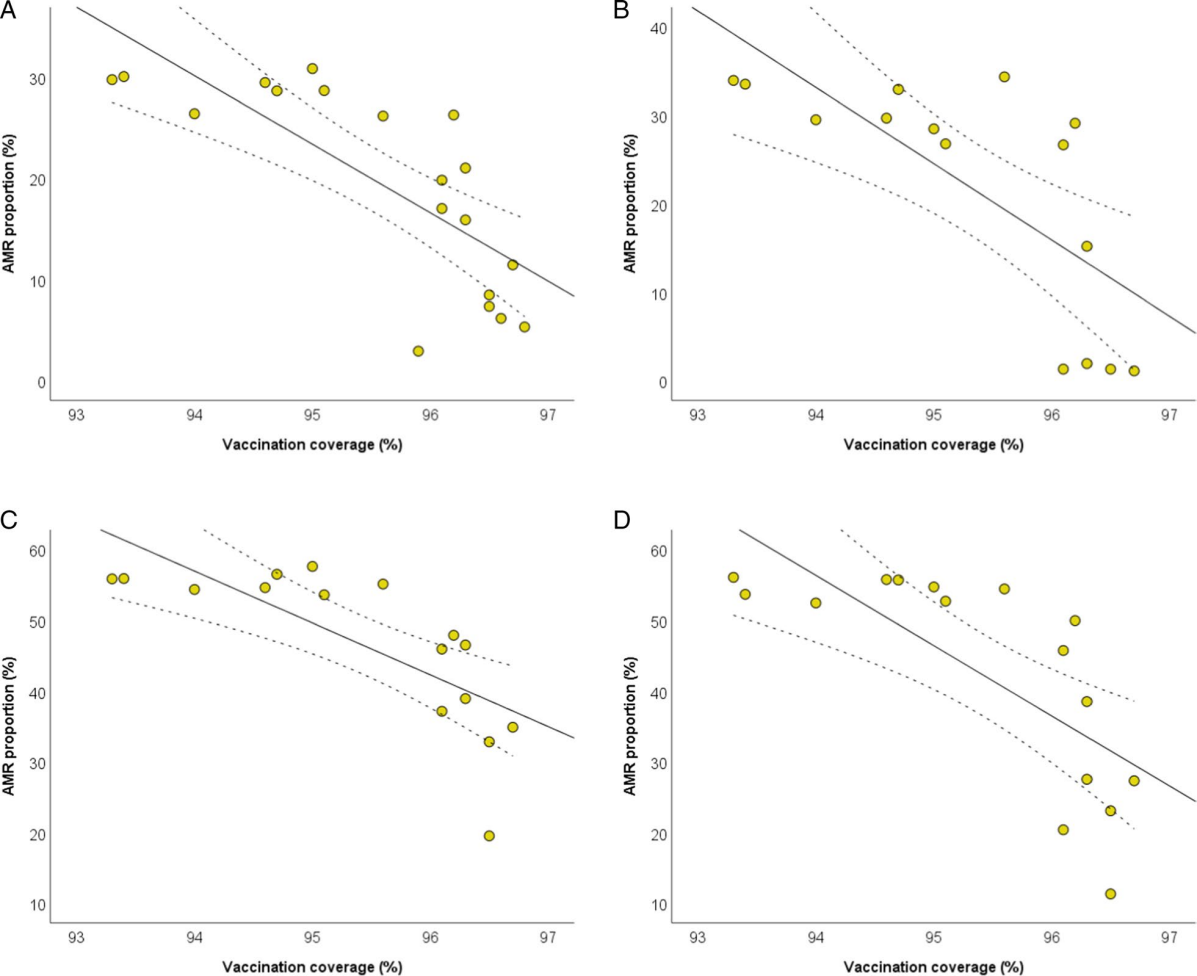
The relationship between polio vaccination coverage and AMR. Proportions of *Escherichia coli* resistant to third generation cephalosporins (**A**); *K. pneumoniae* resistant to carbapenems (**B**), third generation cephalosporins (**C**), and fluoroquinolones (**D**). The plots show linear regression lines and their 95%CI (dotted lines)

Figure 5 shows the scatter plots of the inverse relationships of polio vaccination coverage with AMR proportions of *E. coli*, *K. pneumoniae*, and *P*. aeruginosa. Adjusted linear regression analysis confirmed the inverse relationships of polio vaccination coverage with the proportions of *E. coli* resistant to third generation cephalosporins (β =  − 4.334; SE = 1.457; *p* = 0.009), and *K. pneumoniae* resistant to third generation cephalosporins (β =  − 5.921; SE = 2.090; *p* = 0.015). All the results obtained by applying linear regression models are reported in the Additional file 6.

## Discussion

Although formal studies investigating the impact of vaccination on reducing AMR are still scarce, it is plausible to hypothesize an important contribution of vaccines by reducing antibiotic use and therefore selection pressure on pathogens. Thus, in the present study, we performed an ecological analysis of Italian data to evaluate the relationship between vaccination coverage in children and AMR proportions. Our findings proposed an inverse relationship between vaccination coverage and AMR proportion, especially for some combinations of vaccines, pathogens, and antimicrobials. The relationship was more pronounced for pertussis, diphtheria, tetanus, and polio vaccines; the rest of the vaccines (Hib, MMR, and varicella) also showed some less marked correlations, which however were not significant after correction for multiple comparisons.

This means that AMR proportions decreased with increasing vaccination coverage. However, the ecological nature of our investigation, as well as the unmeasured impact of other factors, suggest caution when interpreting these findings. In fact, the observed relationships did not satisfy the well-known Hill's criteria: a set of nine principles to be satisfied when establishing epidemiological evidence of a causal relationship (i.e., strength, consistency, specificity, temporality, biological gradient, plausibility, coherence, experiment, and analogy). Thus, vaccination coverage does not directly cause AMR, but it may be related to other factors affecting it. Our results, however, supported—at least partially—the hypothesis that the proportions of antimicrobial-resistant pathogens could be reduced by increasing vaccination coverages in childhood. At the moment, however, this remains a concept that should be further investigated by designing and conducting specific analytical and experimental studies.

There are several mechanisms by which vaccines can prevent AMR of non-target pathogens, including the ability to reduce inappropriate antibiotic use and the lower probability of selecting resistant genes than antibiotics. If for the first mechanism, there are a lot of examples showing the reduction of inappropriate antibiotic prescriptions by increasing viral vaccination coverage [10, 21–24], evidence supporting the second mechanism is still scarce. As vaccines are used prophylactically, however, it has been proposed that resistance-conferring mutations are less likely to develop and spread. In addition, vaccines contrast AMR in several ways (both directly and indirectly), making it necessary for the pathogen to undergo multiple mutations to become resistant [9].

Despite the lack of clinical evidence on the impact of childhood vaccination on AMR, some general consideration can be done. According to the Action Framework Leveraging Vaccines to Reduce Antibiotic Use and Prevent Antimicrobial Resistance, published by the WHO, the most direct way by which vaccination could prevent and control AMR is by reducing the incidence of infections from antimicrobial-resistant pathogens [25]. In particular, vaccines against *S. pneumoniae*, Hib, *Salmonella Typhi*, *Bordetella pertussis*, tuberculosis, and *Neisseria meningitidis* can prevent infections from antimicrobial-resistant forms of these pathogens [25]. In our analysis, for example, AMR proportions decreased with increasing pertussis vaccination coverage, especially for *E. coli* and *K. pneumoniae*. In fact, pertussis vaccination coverage was inversely related to the proportions of *E. coli* resistant to fluoroquinolones and third generation cephalosporins, and with the proportions of *K. pneumoniae* resistant to carbapenems and third generation cephalosporins, even considering the effect of covariates (i.e., the number of isolates tested and specific antimicrobial use over the years). A strong inverse relationship was also evident with the proportion of *P. aeruginosa* resistant to piperacillin and tazobactam. It should be noted, however, that similar results were obtained for diphtheria, tetanus, and polio vaccinations as well. This is one of the motivations that still makes it challenging to measure the magnitude and direct effect of vaccines on AMR. Vaccines mentioned above, in fact, are often administered together (i.e., DTaP or the hexavalent formulation), resulting in similar vaccination coverage. For this reason, it is difficult to understand which of them could be helpful in the fight against AMR.

During a recent survey, Italian experts expressed their opinions on the impact of existing vaccines in limiting AMR: about half of them agreed that vaccinations play a role against AMR, attributing the greatest importance to bacterial vaccination, such as pertussis and meningococcal vaccines [26]. Despite not being directly included in the WHO Action Plan at present, the WHO supports the use of vaccines in preventing AMR and encourages further and deeper research to confirm this hypothesis. For example, pertussis infection heals without any consequences and is often underdiagnosed among adults without chronic illnesses [27]. As a consequence, however, it is likely that most patients with pertussis receive at least one antimicrobial prescription if symptoms persist [27]. Since antimicrobials are often empirically prescribed without any etiological diagnosis, the indirect reduction of antimicrobial use is another key benefit of vaccination.

For some of the abovementioned vaccines, the specific impact on AMR has been estimated, for example S. pneumoniae and Hib [28]. The latter was probably the first bacterial vaccine that also showed efficacy in reducing antimicrobial use and hence the development of AMR. Before the introduction of conjugate vaccines, in fact, Hib was a harmful pathogen in infants and children, with high incidence rates in many countries of the world [29]. This was accompanied by a high proportion of Hib β-lactam resistance [30, 31]. Yet, the introduction of Hib conjugate vaccines led to the reduction of Hib incidence rates [32–34] and of β-lactamase-positive strains [35, 36]. The implementation of viral vaccines also proved to be useful for reducing antibiotic use. While our previous study suggested how increasing influenza vaccination coverage could help in the fight against AMR [14], there is no evidence regarding the potential effect of varicella vaccine. Yet, it has been reported that antibiotics are regularly prescribed for the management of patients with varicella, often without any mention of complications or microbiological confirmation [37, 38]. For this reason, it is possible to assume that even varicella vaccine could be effective in reducing the incidence of infections sustained by antimicrobial-resistant pathogens [39]. A similar effect could be attributed to other viral vaccines, such as measles-containing vaccines. In fact, measles vaccine is expected to reduce antibiotic use against secondary bacterial complications, although no exact figures are available [25]. Despite these premises, in our study, coverages for Hib, varicella, and MMR vaccines showed inverse but less marked correlations with AMR proportions, which were not significant after correction for multiple comparisons. For this reason, further studies are needed to clarify whether even these vaccinations could be effective in reducing antibiotic use and AMR.

Overall, our results—along with what has been shown previously—support the hypothesis that AMR proportions could be reduced by increasing vaccination coverage in children. However, caution is required due to the ecological design and additional weakness of our study. Our analysis, in fact, did not focus on individual data, but only on Italian indicators over the last 2 decades. Thus, results are prone to biases and misinterpretations that do not allow us to evaluate a causal effect between childhood vaccination and AMR [40]. Moreover, the observed relationships were generally weak, though statistically significant. This was not surprising since other factors (e.g., antibiotic use and appropriateness) have a greater impact on AMR than vaccines. Data quality represents another limitation, since certain inaccuracies in vaccination coverage estimates cannot be completely excluded due to different characteristics and functionalities of immunization registries implemented at regional level [15]. In Italy, in fact, the national health system is decentralized and requires efforts to guarantee the interoperability between different systems adopted at regional level. It is worth noting that some vaccines exhibited constant high coverages over the years, while others had extremely varied values. On the one hand, high values of coverage for some vaccines did not allow us to estimate how much AMR could prevented by increasing vaccination coverages. On the other hand, the extremely varied values observed for other vaccines might affect their specific results. Moreover, there is a lack of data on vaccination coverage against Pneumococcus, although the pneumococcal conjugate vaccine has been introduced in Italy with the National Plan for Vaccine Prevention 2005–2007. Thus, further analysis, even considering data from other European countries, could help better understand the relationship between vaccination coverage and AMR proportions. It is also worth underlining that data on AMR proportions were obtained through the activity of a network of sentinel national laboratories referring to the ECDC. Data coverage in this case is still low—approximately 50%—despite an increase over time [18]. To partially address this issue, we adjusted our analysis by including the number of isolates tested as a covariate of the multivariable linear regression models. Data integration represents another limitation of our study, since measures and indicators we used were collected from different samples of the Italian population (i.e., vaccination coverages in children and AMR proportions in the overall population). For this reason, our findings do not reflect relationships observed at individual level. Finally, the lack of data on potential confounding and/or mediating factors kept us from taking into account mechanisms influencing or supporting the observed relationships [40]. For example, differences in vaccine supply, social and cultural background, and health conditions at individual and community level could confound the analyses. Moreover, reliable data on age-specific antibiotic use, incidence of infections sustained by antimicrobial-resistant pathogens, medications, and hospitalizations due to these infections could be useful to improve the interpretation of our findings.

Based on these considerations, our study represents a first step in the path of confirming the complementary role of childhood vaccination coverage against AMR. Given the paucity of scientific data on this topic at present, it is still important to investigate how AMR proportions change with changing vaccination coverage. Due to the main limitations described above, it was not our intention to estimate the proportion of AMR that could be prevented by increasing vaccination coverages in children. However, evaluating the role of children vaccination is still an emerging field of research that could be crucial to identify alternative strategies to fight AMR. This becomes even more important if considering the current Italian epidemiological scenario, which requires that all potential interventions against AMR should be evaluated and considered. Although Italy is among the countries most affected by AMR in Europe, further analysis of data from other European countries should be encouraged given the importance of AMR globally. Another future direction in the field, instead, should be the research on mechanisms underpinning the observed relationships, as well as the influence of potential confounding and mediating factors. Since our results do not prove that childhood vaccination can reduce AMR proportions, further studies—especially with analytical and/or experimental designs—are urgently needed in order to understand and predict how increasing childhood vaccination coverage will affect AMR.

## Conclusions

Our analysis of Italian data suggested an inverse relationship between vaccination coverage in children and AMR proportions in the overall population. Although these findings are generally in accordance with the hypothesis that high vaccination coverages reduce antimicrobial prescriptions and inappropriate antimicrobial use, further efforts should be encouraged to elucidate mechanisms supporting the observed relationships. Where justified by studies designed ad hoc for this purpose, this evidence could be used to inform global and national actions plans dedicated to AMR control, by including vaccination as an additional strategy against AMR.

## Supplementary Information

**Additional file 1.** Trends of vaccination coverages in Italy from 2000 to 2020. Data are publicly available on the website www.epicentro.iss.it/vaccini/dati_Ita.

**Additional file 2.** Trends of AMR proportions for specific combinations of pathogens and antibiotics. Data are publicly available from the Surveillance Atlas of Infectious Diseases of the ECDC.

**Additional file 3.** Linear regressions of the association between pertussis vaccination coverage and antimicrobial resistance, adjusted for number of isolates tested and antimicrobial use.

**Additional file 4.** Linear regressions of the association between diphtheria vaccination coverage and antimicrobial resistance, adjusted for number of isolates tested and antimicrobial use.

**Additional file 5.** Linear regressions of the association between tetanus vaccination coverage and antimicrobial resistance, adjusted for number of isolates tested and antimicrobial use.

**Additional file 6.** Linear regressions of the association between polio vaccination coverage and antimicrobial resistance, adjusted for number of isolates tested and antimicrobial use.

## Data Availability

The datasets used and/or analysed during the current study are available from the corresponding author on reasonable request.

## Abbreviations

AMR: Antimicrobial resistance
ECDC: European Centre for Disease Prevention and Control
ISS: Istituto Superiore di Sanità
Hib: *Haemophilus influenzae* Type B
MMR: Measles, Mumps, and Rubella
DTaP: Diphtheria-tetanus-pertussis vaccine
MRSA: *S. aureus* Resistant to methicillin
TESSy: European Surveillance System
ATC: Anatomical Therapeutic Chemical
DDD: Defined Daily Dose
ISTAT: Istituto Nazionale di Statistica
SD: Standard deviation
SE: Standard Error
