# Supplementary material for: Vaccination coverage in Italian children and antimicrobial resistance: an ecological analysis
Source: Antimicrob Resist Infect Control. 2022 Nov 9;11:136. doi: 10.1186/s13756-022-01173-0 (PMC9648027; doi:10.1186/s13756-022-01173-0)
Supplement: Supplementary file 1 — Additional file 1. Trends of vaccination coverages in Italy from 2000 to 2020. Data are publicly available on the website www.epicentro.iss.it/vaccini/dati_Ita. [file 13756_2022_1173_MOESM1_ESM.docx]

**Additional File 1.** Trends of vaccination coverages in Italy from 2000 to 2020. Data are publicly available on the website www.epicentro.iss.it/vaccini/dati_Ita.

**
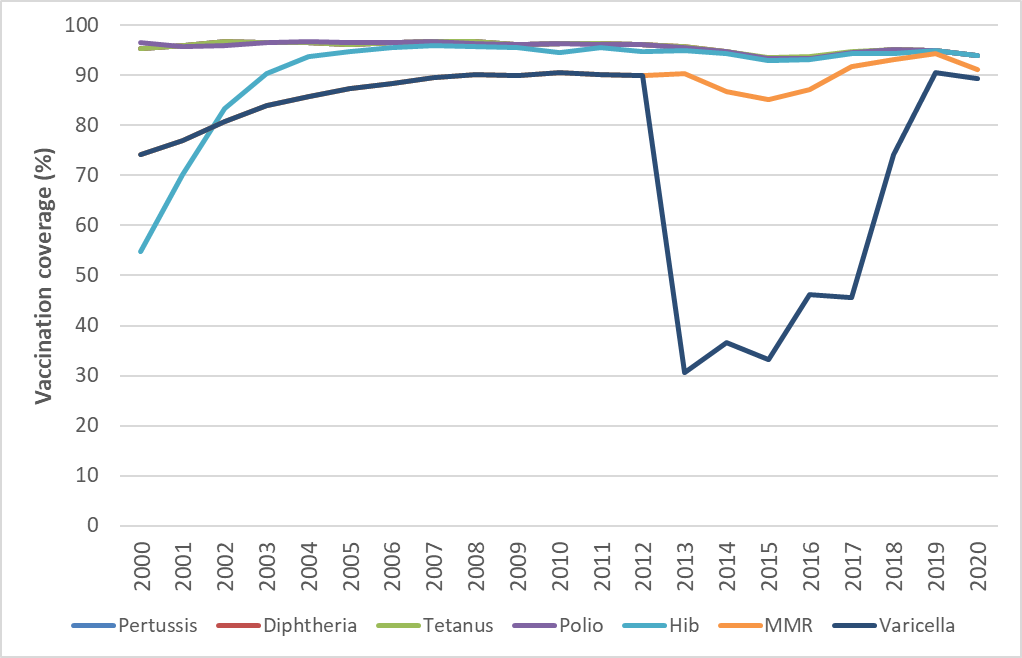
**
