# Supplementary material for: Vaccination coverage in Italian children and antimicrobial resistance: an ecological analysis
Source: Antimicrob Resist Infect Control. 2022 Nov 9;11:136. doi: 10.1186/s13756-022-01173-0 (PMC9648027; doi:10.1186/s13756-022-01173-0)
Supplement: Supplementary file 2 — Additional file 2. Trends of AMR proportions for specific combinations of pathogens and antibiotics. Data are publicly available from the Surveillance Atlas of Infectious Diseases of the ECDC. [file 13756_2022_1173_MOESM2_ESM.docx]

**Additional File 2.** Trends of AMR proportions for specific combinations of pathogens and antibiotics. Data are publicly available from the Surveillance Atlas of Infectious Diseases of the ECDC.

**
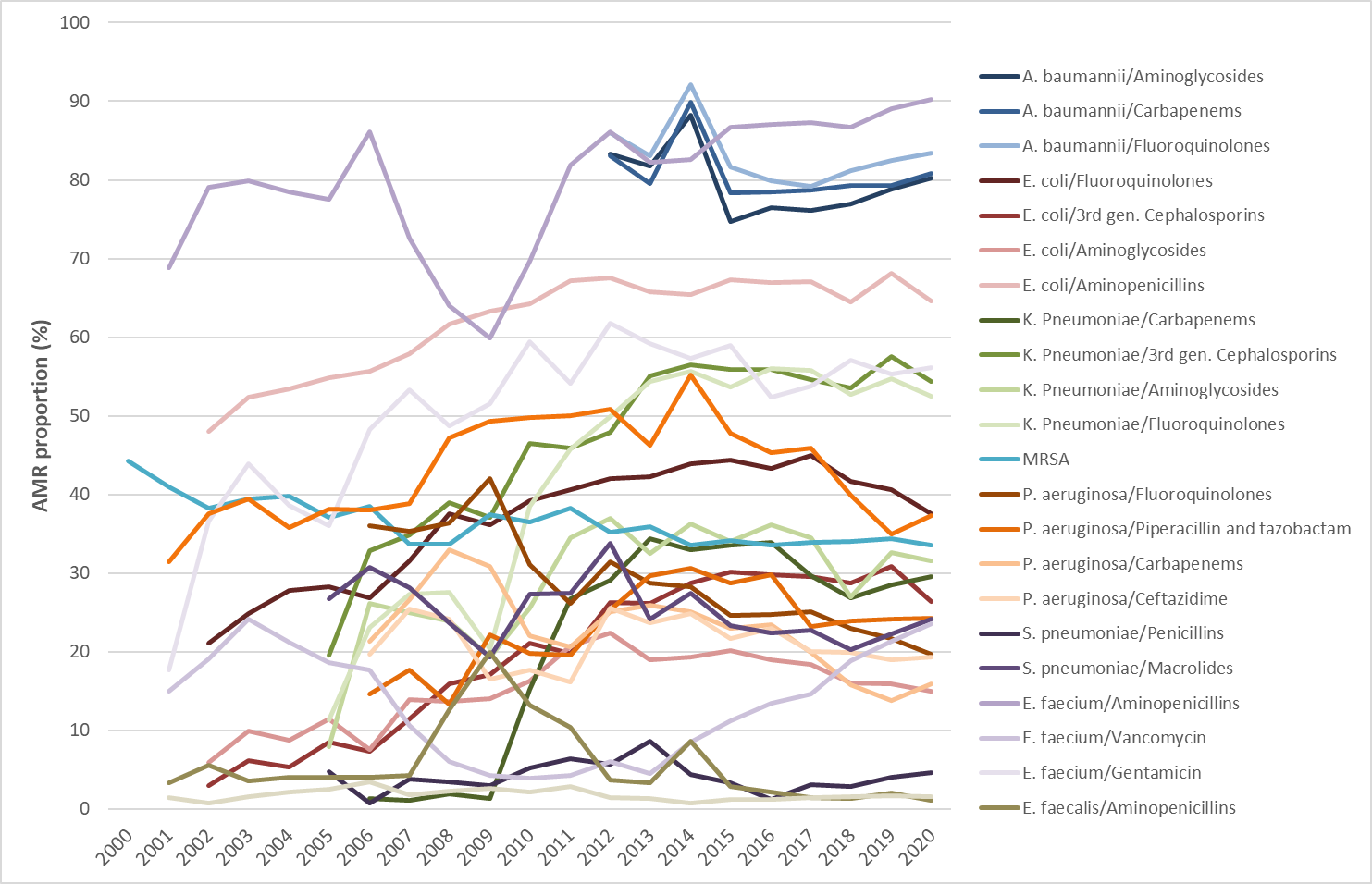
**
