# Supplementary material for: Vaccination coverage in Italian children and antimicrobial resistance: an ecological analysis
Source: Antimicrob Resist Infect Control. 2022 Nov 9;11:136. doi: 10.1186/s13756-022-01173-0 (PMC9648027; doi:10.1186/s13756-022-01173-0)
Supplement: Supplementary file 3 — Additional file 3. Linear regressions of the association between pertussis vaccination coverage and antimicrobial resistance, adjusted for number of isolates tested and antimicrobial use. [file 13756_2022_1173_MOESM3_ESM.docx]

**Additional File 3.** Linear regressions of the association between pertussis vaccination coverage and antimicrobial resistance, adjusted for number of isolates tested and antimicrobial use.

| Isolates | Antibiotics | β | SE | p-value |
| --- | --- | --- | --- | --- |
| E. coli resistant to Fluoroquinolones | Vaccination coverage | -2.823 | 1.176 | 0.030 |
|  | Number of isolates | 0.002 | 0.001 | 0.012 |
|  | Antibiotic use | 7.528 | 2.152 | 0.003 |
| E. coli resistant to 3rd gen. Cephalosporins | Vaccination coverage | -4.620 | 1.416 | 0.005 |
|  | Number of isolates | 0.003 | 0.001 | 0.012 |
|  | Antibiotic use | 18.373 | 9.740 | 0.079 |
| K. pneumoniae resistant to Carbapenems | Vaccination coverage | -4.247 | 1.767 | 0.038 |
|  | Number of isolates | 0.001 | 0.001 | 0.993 |
|  | Antibiotic use | 11.023 | 2.862 | 0.003 |
| K. pneumoniae resistant to 3rd gen. Cephalosporins | Vaccination coverage | -5.456 | 2.170 | 0.027 |
|  | Number of isolates | 0.002 | 0.001 | 0.164 |
|  | Antibiotic use | 16.152 | 16.934 | 0.359 |
| P. aeruginosa resistant to Piperacillin and tazobactam | Vaccination coverage | -4.131 | 0.974 | 0.001 |
|  | Number of isolates | -0.002 | 0.001 | 0.051 |
|  | Antibiotic use | 5.024 | 2.384 | 0.059 |
