# Supplementary material for: Vaccination coverage in Italian children and antimicrobial resistance: an ecological analysis
Source: Antimicrob Resist Infect Control. 2022 Nov 9;11:136. doi: 10.1186/s13756-022-01173-0 (PMC9648027; doi:10.1186/s13756-022-01173-0)
Supplement: Supplementary file 4 — Additional file 4. Linear regressions of the association between diphtheria vaccination coverage and antimicrobial resistance, adjusted for number of isolates tested and antimicrobial use. [file 13756_2022_1173_MOESM4_ESM.docx]

**Additional File 4.** Linear regressions of the association between diphtheria vaccination coverage and antimicrobial resistance, adjusted for number of isolates tested and antimicrobial use.

| Isolates | Antibiotics | β | SE | p-value |
| --- | --- | --- | --- | --- |
| E. coli resistant to Fluoroquinolones | Vaccination coverage | -2.856 | 1.190 | 0.030 |
|  | Number of isolates | 0.002 | 0.001 | 0.012 |
|  | Antibiotic use | 7.735 | 2.147 | 0.003 |
| E. coli resistant to 3rd gen. Cephalosporins | Vaccination coverage | -4.648 | 1.450 | 0.006 |
|  | Number of isolates | 0.003 | 0.001 | 0.012 |
|  | Antibiotic use | 19.694 | 9.826 | 0.063 |
| K. pneumoniae resistant to Carbapenems | Vaccination coverage | -4.734 | 2.080 | 0.039 |
|  | Number of isolates | 0.001 | 0.001 | 0.712 |
|  | Antibiotic use | 10.123 | 2.653 | 0.004 |
| K. pneumoniae resistant to 3rd gen. Cephalosporins | Vaccination coverage | -5.404 | 2.258 | 0.034 |
|  | Number of isolates | 0.002 | 0.001 | 0.171 |
|  | Antibiotic use | 17.524 | 17.214 | 0.329 |
| P. aeruginosa resistant to Piperacillin and tazobactam | Vaccination coverage | -3.885 | 1.085 | 0.004 |
|  | Number of isolates | -0.001 | 0.001 | 0.415 |
|  | Antibiotic use | 5.127 | 2.684 | 0.069 |
