# Supplementary material for: Vaccination coverage in Italian children and antimicrobial resistance: an ecological analysis
Source: Antimicrob Resist Infect Control. 2022 Nov 9;11:136. doi: 10.1186/s13756-022-01173-0 (PMC9648027; doi:10.1186/s13756-022-01173-0)
Supplement: Supplementary file 5 — Additional file 5. Linear regressions of the association between tetanus vaccination coverage and antimicrobial resistance, adjusted for number of isolates tested and antimicrobial use. [file 13756_2022_1173_MOESM5_ESM.docx]

**Additional File 5.** Linear regressions of the association between tetanus vaccination coverage and antimicrobial resistance, adjusted for number of isolates tested and antimicrobial use.

| Isolates | Antibiotics | β | SE | p-value |
| --- | --- | --- | --- | --- |
| E. coli resistant to Fluoroquinolones | Vaccination coverage | -3.059 | 1.260 | 0.028 |
|  | Number of isolates | 0.002 | 0.001 | 0.014 |
|  | Antibiotic use | 7.761 | 2.139 | 0.002 |
| E. coli resistant to 3rd gen. Cephalosporins | Vaccination coverage | -4.944 | 1.538 | 0.006 |
|  | Number of isolates | 0.003 | 0.001 | 0.015 |
|  | Antibiotic use | 19.476 | 9.812 | 0.066 |
| K. pneumoniae resistant to Carbapenems | Vaccination coverage | -4.380 | 2.095 | 0.043 |
|  | Number of isolates | 0.002 | 0.001 | 0.458 |
|  | Antibiotic use | 11.202 | 2.906 | 0.003 |
| K. pneumoniae resistant to 3rd gen. Cephalosporins | Vaccination coverage | -5.744 | 2.403 | 0.033 |
|  | Number of isolates | 0.001 | 0.001 | 0.319 |
|  | Antibiotic use | 15.024 | 16.123 | 0.726 |
| P. aeruginosa resistant to Piperacillin and tazobactam | Vaccination coverage | -4.184 | 1.138 | 0.003 |
|  | Number of isolates | -0.001 | 0.001 | 0.357 |
|  | Antibiotic use | 5.001 | 2.691 | 0.079 |
