# Supplementary material for: Vaccination coverage in Italian children and antimicrobial resistance: an ecological analysis
Source: Antimicrob Resist Infect Control. 2022 Nov 9;11:136. doi: 10.1186/s13756-022-01173-0 (PMC9648027; doi:10.1186/s13756-022-01173-0)
Supplement: Supplementary file 6 — Additional file 6. Linear regressions of the association between polio vaccination coverage and antimicrobial resistance, adjusted for number of isolates tested and antimicrobial use. [file 13756_2022_1173_MOESM6_ESM.docx]

**Additional File 6.** Linear regressions of the association between polio vaccination coverage and antimicrobial resistance, adjusted for number of isolates tested and antimicrobial use.

| Isolates | Antibiotics | β | SE | p-value |
| --- | --- | --- | --- | --- |
| E. coli resistant to 3rd gen. Cephalosporins | Vaccination coverage | -4.334 | 1.457 | 0.009 |
|  | Number of isolates | 0.003 | 0.001 | 0.007 |
|  | Antibiotic use | 18.562 | 10.100 | 0.086 |
| K. pneumoniae resistant to 3rd gen. Cephalosporins | Vaccination coverage | -5.921 | 2.090 | 0.015 |
|  | Number of isolates | 0.002 | 0.001 | 0.166 |
|  | Antibiotic use | 15.777 | 16.201 | 0.349 |
